# Relationship between transforming growth factor-β1 and type 2 diabetic nephropathy risk in Chinese population

**DOI:** 10.1186/s12881-018-0717-3

**Published:** 2018-11-20

**Authors:** Tianbiao Zhou, Hong-Yan Li, Hongzhen Zhong, Zhiqing Zhong

**Affiliations:** 1 Department of Nephrology, the Second Affiliated Hospital of Shantou University Medical College, No 69 Dongsha Road, Shantou, 515041 China; 2 Department of Nephrology, Huadu District People’s Hospital of Guangzhou, Southern Medical University, Guangzhou, 510800 China

**Keywords:** Type 2 diabetic nephropathy (T2DN), Diabetes mellitus (DM), Transforming growth factor-β1, T869C, Gene polymorphism, Meta-analysis

## Abstract

**Background:**

Diabetes mellitus (DM) is divided into four different etiological categories: type 1 DM (T1DM), type 2 DM (T2DM), other specific types, and gestational DM. One severe complication of T2DM is type 2 diabetic nephropathy (T2DN). The possible association of serum transforming growth factor-β1 (TGF-β1) levels and the TGF-β1 T869C gene polymorphism with patient susceptibility to T2DN in Chinese population is unclear at present. This study was conducted to assess these relationships in Chinese population by a meta-analysis.

**Methods:**

Association reports were searched and pulled from the Cochrane Library, the China Biological Medicine Database (CBM), and PubMed on March 1, 2018, and eligible studies were selected and used for calculations. The results were expressed as weighted mean differences (MD) for continuous data. Odds ratios (OR) were used to express the results for dichotomous data. Additionally, 95% confidence intervals (CI) were calculated.

**Results:**

Forty-eight reports for the relationship between serum TGF-β1 levels and the risk of T2DN and 13 studies on the association of the TGF-β1 T869C gene polymorphism with susceptibility to T2DN in Chinese population were retrieved from this study. Serum TGF-β1 levels in the T2DM group were higher than those in the normal control group (MD = 17.30, 95% CI: 12.69–21.92, *P* < 0.00001). The serum TGF-β1 level in the T2DN group was significantly higher than that in the normal control group (MD = 70.03, 95% CI: 60.81–79.26, *P <* 0.00001;). The serum TGF-β1 level in the T2DN group was significantly higher than that in the T2DM group (MD = 56.18, 95% CI: 46.96–65.39, *P <* 0.00001). Serum TGF-β1 levels in T2DM patients with microalbuminuria were increased when compared with those in T2DM patients with normoalbuminuria. Furthermore, serum TGF-β1 levels in T2DM patients with macroalbuminuria were increased when compared with those in T2DM patients with microalbuminuria. The TGF-β1 T allele, TT allele and CC genotype were associated with T2DN susceptibility in Chinese population (T: OR = 0.74, 95% CI: 0.59–0.92, *P* = 0.007; TT: OR = 0.55, 95% CI: 0.31–0.96, *P* = 0.04; CC: OR = 1.38, 95% CI: 1.14–1.67, *P* = 0.001).

**Conclusions:**

High levels of TGF-β1 are associated with susceptibility to T2DM, T2DN and the progression of proteinuria in T2DN patients in Chinese population. Further, the TGF-β1 T allele, and TT genotype were protective factors against the onset of T2DN and CC genotype was a risk factor for the susceptibility of T2DN in Chinese populations.

## Background

Transforming growth factor beta1 (TGF-β1) is one of the pro-fibrotic cytokines and is thought to be the primary mediator driving the progression of fibrosis, glomerulosclerosis and especially mesangial cell phenotype transformation in diabetic nephropathy (DN) [1, 2]. TGF-β1 directly stimulates the transcription of extracellular matrix (ECM). Increased TGF-β1 is reported to be associated with DN disease [3–5]. Gene polymorphisms of TGF-β1 can affect the activity of TGF-β1. The TGF-β1 T869C gene polymorphism is one of the most important gene polymorphisms that affects the protein expression of TGF-β1 [6]. Gene polymorphisms have been reported to be associated with some diseases [7–9]. However, there are conflicting reports on the association of the TGF-β1 T869C polymorphism with T2DN susceptibility [10–13].

Diabetes mellitus (DM), characterized by elevated levels of blood glucose, is a complex and heterogeneous, chronic metabolic disease [14]. DM is the leading cause of morbidity and mortality worldwide and is a major global health problem [15, 16]. DM is divided into four different etiological categories: type 1 DM (T1DM), type 2 DM (T2DM), other specific types, and gestational DM. The main characteristic of T2DM is insulin resistance, often followed by the failure of pancreatic β-cells. Recent data indicate that morbidity and mortality among diabetic patients are increased [14]. One severe complication of T2DM is type 2 diabetic nephropathy (T2DN), which is characterized by hypertension, albuminuria, and a progressive decline in glomerular filtration rate, developing into end-stage renal disease [17, 18]. There is increasing evidence showing that TGF-β1 takes part in the pathogenesis of T2DN [19–21].

In this study, we assessed the association between TGF-β1 levels and T2DN risk, and the association of the TGF-β1 T869C gene polymorphism with the susceptibility to T2DN in Chinese population, by a meta-analysis method.

## Methods

### Search strategy

The electronic databases of the Cochrane Library, the China Biological Medicine Database (CBM), and PubMed were searched on March 1, 2018, and relevant studies were retrieved. The retrieval strategy of “(transforming growth factor-β1 OR TGF-β1) AND (diabetic nephropathy OR diabetic kidney disease)” was entered and searched in these databases. Additional investigations were extracted from the references cited in articles retrieved in this search.

### Inclusion and exclusion criteria

#### Inclusion criteria

(1) Each study had at least two comparison groups (case group vs. control group); (2) The outcome in patients had to be T2DN; (3) Each study should show data on the TGF-β1 level and/or the TGF-β1 T869C genotype distribution.

#### Exclusion criteria

(1) Editorials, review articles, case reports; (2) Study results not showing the TGF-β1 level or the TGF-β1 T869C gene polymorphism to disease; (3) Multiple publications from the same study group; (4) Study not conducted in Chinese population.

### Data extraction and synthesis

The information was extracted from each eligible report by two authors independently: the surname of the first author, the publication year, the country of the study or ethnicity, the TGF-β1 levels, the number of patients or controls, and the number of subjects in case groups and control groups for TGF-β1 genotypes.

### Statistical analysis

Cochrane Review Manager Version 5 software (Cochrane Library, UK) was used to calculate the available data from each investigation. The fixed effects model was used to calculate the pooled statistic. However, a random effects model was used to assess the relationship when the *P* value of the heterogeneity test was less than 0.1. The results were expressed as weighted mean differences (MD) for continuous data, and odds ratios (OR) were used to express the results for dichotomous data. Additionally, 95% confidence intervals (CI) were also counted. *P* < 0.05 was required for statistical significance for the pooled OR. *I*^*2*^ was used to test the heterogeneity among the included investigations. The Egger regression asymmetry test [22] and the Begg adjusted-rank correlation test [23] were used to test the publication bias, and *P* < 0.10 was considered significant.

## Results

### Study characteristics

Forty-five reports [24–68] were included for the meta-analysis of the relationship between TGF-β1 level and T2DN risk in Chinese population (Table 1). One report [67] was published in English and other reports were published in Chinese.

**Table 1:** General characteristics of the included studies for TGF-β1 levels in T2DN in this meta-analysis

First author, year	Country	According to	Case			Control		
		UAER or UACR	Mean	SD	N	Mean	SD	N
Ju HB 2000	China	Normoalbuminuria	35.02	6.7	14	23.95	8.01	15
		Microalbuminuria	39.31	5.35	18	23.95	8.01	15
		Macroalbuminuria	58.58	9.56	13	23.95	8.01	15
Wang YJ 2002	China	Normoalbuminuria	147.03	22.57	34	136.97	37.96	35
		Macroalbuminuria	170.65	18.74	31	136.97	37.96	35
Li WM 2004	China	Normoalbuminuria	58.91	11.03	46	47.25	6.22	48
		Macroalbuminuria	387.45	82.06	48	47.25	6.22	48
Li ZJ 2004	China	Normoalbuminuria	146	22	36	131	36	40
		Macroalbuminuria	172	19	44	131	36	40
Jiang ZL 2005	China	Normoalbuminuria	428.3	43.7	29	412.5	58.4	35
		Microalbuminuria	578.5	69.4	27	412.5	58.4	35
		Macroalbuminuria	683.4	84.3	28	412.5	58.4	35
Li ZZ 2005	China	Normoalbuminuria	41	15.57	27	10.04	5.33	18
		Microalbuminuria	66.35	18.04	12	10.04	5.33	18
		Macroalbuminuria	53.31	15.64	18	10.04	5.33	18
Zhou Y 2005	China	Normoalbuminuria	31.12	12.39	30	29.4	10.62	30
		Microalbuminuria	79.63	15.96	30	29.4	10.62	30
		Macroalbuminuria	136.6	21.45	30	29.4	10.62	30
Jing CY 2005	China	Normoalbuminuria	31.16	14.23	31	24.58	12.61	20
		Microalbuminuria	48.2	18.3	25	24.58	12.61	20
		Macroalbuminuria	62.12	21.3	23	24.58	12.61	20
Wei YS 2005	China	NR	41.57	10.55	91	25.46	7.88	105
Li HP 2006	China	Normoalbuminuria	147.02	20.57	108	131.96	3.84	120
		Macroalbuminuria	170.64	17.72	132	131.96	3.84	120
Tao SP 2006	China	Normoalbuminuria	147	23	28	132	36	25
		Macroalbuminuria	172	18	34	132	36	25
Meng T 2006	China	Normoalbuminuria	217.7	126	28	84.5	23.4	30
		Microalbuminuria	288.2	109.4	24	84.5	23.4	30
		Macroalbuminuria	345.5	118.2	22	84.5	23.4	30
Xie HF 2006	China	Normoalbuminuria	42.1	9.3	60	35.9	8.1	30
		Macroalbuminuria	61.8	11.2	45	35.9	8.1	30
Qian YX 2006	China	Normoalbuminuria	146	22	48	131	36	60
		Macroalbuminuria	172	19	23	131	36	60
Fu CX 2007	China	Normoalbuminuria	36.2	8.8	34	34.4	8.2	35
		Microalbuminuria	69.4	12.8	31	34.4	8.2	35
Du JW 2007	China	Normoalbuminuria	179.16	13.13	20	68.47	31.75	19
		Microalbuminuria	192.66	57.25	21	68.47	31.75	19
		Macroalbuminuria	582.04	211.25	20	68.47	31.75	19
Zhang WJ 2007	China	Normoalbuminuria	23.35	3.7	36	20.35	3.7	40
		Microalbuminuria	41.31	4.3	45	20.35	3.7	40
		Macroalbuminuria	55.28	6.8	45	20.35	3.7	40
Lai X 2007	China	Normoalbuminuria	89.65	28.33	27	31.46	9.07	43
		Microalbuminuria	121.02	32.36	21	31.46	9.07	43
		Macroalbuminuria	211.69	69.83	17	31.46	9.07	43
Lin YH 2007	China	Normoalbuminuria	97.24	18.6	19	58.36	13.72	23
		Macroalbuminuria	136.75	23.48	24	58.36	13.72	23
Zhang SF 2007	China	Microalbuminuria	21.188	20.87	15	6.99	18.57	18
		Macroalbuminuria	13.64	19.44	16	6.99	18.57	18
Zhang WK 2008	China	Normoalbuminuria	23.3	10.1	30	20.3	3.7	26
		Microalbuminuria	41.3	4.2	38	20.3	3.7	26
		Macroalbuminuria	88.2	6.8	32	20.3	3.7	26
Wang YP 2008	China	Normoalbuminuria	35.4	7.1	44	32.5	6.8	35
		Macroalbuminuria	68.2	12.5	32	32.5	6.8	35
Zhang SB 2008	China	NR	121.5	37.2	36	55.2	16.8	30
Li QX 2008	China	Normoalbuminuria	31.9	9.72	26	21.5	6.89	20
		Microalbuminuria	49.6	14.78	23	21.5	6.89	20
		Macroalbuminuria	70.3	26.48	18	21.5	6.89	20
Feng SJ 2008	China	Normoalbuminuria	208.2	110	25	80.62	3.4	38
		Microalbuminuria	293.3	118.5	23	80.62	3.4	38
		Macroalbuminuria	263.5	108.2	18	80.62	3.4	38
Zhang HM 2008	China	Normoalbuminuria	32.52	12.24	40			
		Microalbuminuria	43.61	20.37	48			
Cao B 2009	China	Normoalbuminuria	31.2	5.6	31	17.4	3.4	30
		Microalbuminuria	54.9	7.8	34	17.4	3.4	30
		Macroalbuminuria	78.2	10.3	30	17.4	3.4	30
Li QX 2009	China	Normoalbuminuria	31.9	9.72	26	21.5	6.89	20
		Microalbuminuria	49.6	14.78	23	21.5	6.89	20
		Macroalbuminuria	70.3	26.48	18	21.5	6.89	20
Yang YZ 2010	China	Normoalbuminuria	28.59	3.64	25	21.07	3.48	30
		Macroalbuminuria	43.12	4.62	25	21.07	3.48	30
Feng LM 2010	China	Normoalbuminuria	34.2	7.1	40	32.8	6.4	35
		Macroalbuminuria	69.4	7.2	32	32.8	6.4	35
Wu YJ 2010	China	NG	172.5	20.4	30	125.4	14.6	28
Ye CF 2010	China	Normoalbuminuria	31.36	5.75	37	26.54	5.78	32
		Macroalbuminuria	58.69	9.87	37	26.54	5.78	32
Huang JW 2010	China	Normoalbuminuria	41.85	10.38	29	22.5	5.75	30
		Microalbuminuria	79.51	44.95	32	22.5	5.75	30
		Macroalbuminuria	118.15	59.38	28	22.5	5.75	30
Chen D 2011	China	Normoalbuminuria	129.16	27.08	30	83.32	30.55	60
		Microalbuminuria	162.97	98.58	30	83.32	30.55	60
		Macroalbuminuria	563.46	122.67	30	83.32	30.55	60
Li QX 2011	China	Normoalbuminuria	31.9	9.72	26	21.5	6.89	20
		Microalbuminuria	49.6	14.78	23	21.5	6.89	20
		Macroalbuminuria	70.3	26.48	18	21.5	6.89	20
Zhou ZX 2011	China	Normoalbuminuria	33.12	8.16	50	32.98	7.83	50
		Microalbuminuria	49.21	18.11	56	32.98	7.83	50
He Y 2012	China	Normoalbuminuria	147.01	20.98	48	131.82	36.01	60
		Macroalbuminuria	172.31	19.06	42	131.82	36.01	60
Zhang Y 2012	China	NG	154.8	7.09	28	122.84	6.3	15
Du ZC 2013	China	Normoalbuminuria	18.55	2.67	20	8.97	4.087	18
		Microalbuminuria	19.04	2.87	20	8.97	4.087	18
		Macroalbuminuria	18.12	3.17	21	8.97	4.087	18
Zhang WQ 2014	China	Normoalbuminuria	30.3	4.42	32	24.52	2.81	23
		Microalbuminuria	34.32	4.32	41	24.52	2.81	23
		Macroalbuminuria	58.31	5.16	13	24.52	2.81	23
Liu S 2014	China	Normoalbuminuria	76.8	3.1	30	29.6	2.5	30
		Microalbuminuria	114.8	3.1	30	29.6	2.5	30
		Macroalbuminuria	135.8	5.9	30	29.6	2.5	30
Bao HL 2014	China	Microalbuminuria	75.4	9.2	33	71.2	11.1	36
Feng R 2015	China	Normoalbuminuria	7.58	2.11	22	5.13	1.63	30
		Microalbuminuria	11.89	3.33	29	5.13	1.63	30
		Macroalbuminuria	24.62	6.62	35	5.13	1.63	30
Lv C 2015	China	Normoalbuminuria	27.3	5.45	137	14.98	3.23	131
		Microalbuminuria	51.8	5.72	122	14.98	3.23	131
		Macroalbuminuria	72.97	6.05	68	14.98	3.23	131
Bi FC 2016	China	Normoalbuminuria	5.61	2.08	21	1.79	1.64	20
		Microalbuminuria	8.98	2.26	20	1.79	1.64	20
		Macroalbuminuria	11.39	1.61	20	1.79	1.64	20

*NR*: not report

Eight studies [12, 32, 69–74] reporting the association of the TGF-β1 T869C gene polymorphism with susceptibility to T2DN were included in this study. Two report [69, 74] were published using the English language and the other reports were published using Chinese. The data for the pooled OR were extracted (Table 2). Those 8 investigations contained 1018 patients with T2DN and 941 controls. The average distribution frequency of the TGF-β1 T allele in the T2DN group in Chinese patients was 38.15% and the average frequency in the control group was 44.72%. The average distribution frequency of the TGF-β1 T allele in the case group was lower than that in the control group in Chinese population (Case/Control = 0.85).

**Table 2:** General characteristics of the included studies on TGF-β1 T869C gene polymorphism with T2DN risk in Chinese population

		Case				Control			
Author, Year	Ethnicity	CC	CT	TT	total	CC	CT	TT	total
Wong, 2003	Asian	27	26	5	58	24	24	17	65
Wei, 2005	Asian	31	48	12	91	21	46	25	92
Wei, 2008	Asian	94	128	58	280	72	142	66	280
Chen, 2010	Asian	68	118	46	232	30	63	33	126
Chai, 2009	Asian	19	19	14	52	27	26	5	58
Pan, 2007	Asian	37	34	9	80	34	29	24	87
Rao, 2011	Asian	14	25	6	45	13	32	8	53
Mou, 2011	Asian	88	87	5	180	71	73	36	180

### Association of the TGF-β1 level with T2DN risk

**Fig. 1:**
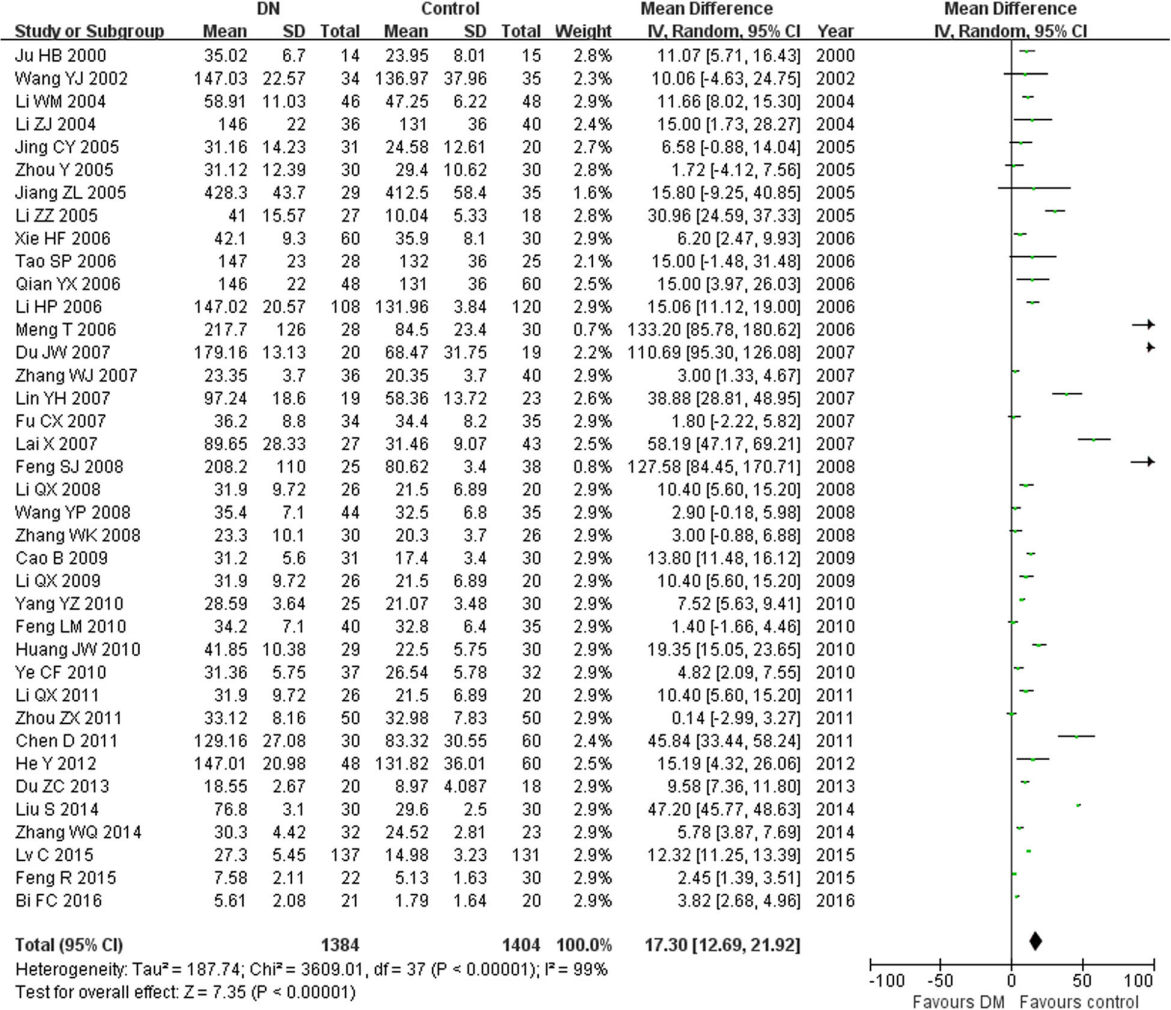
Association of TGF-β1 levels with T2DM susceptibility (T2DM vs. control)

**Fig. 2:**
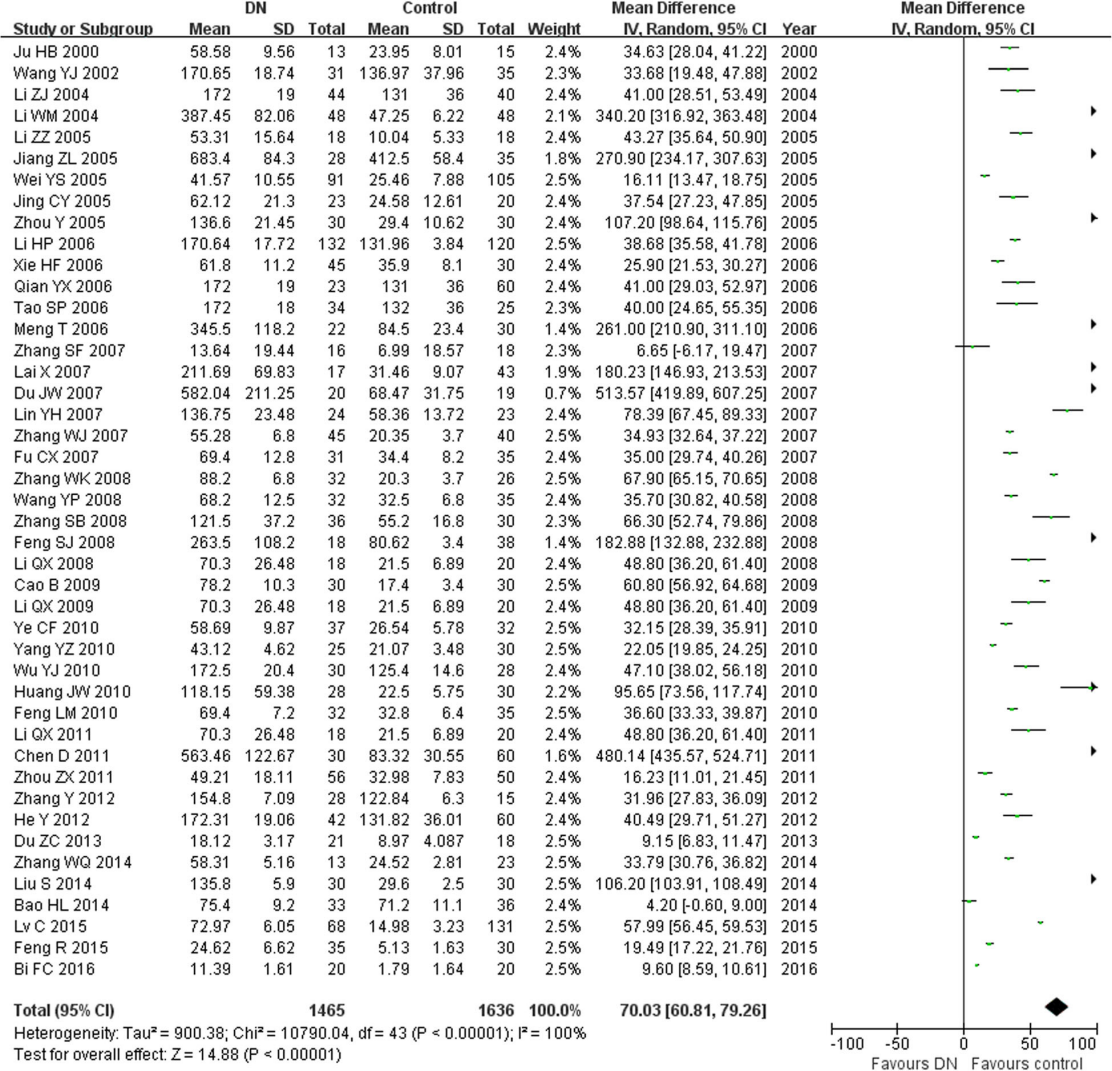
Association of TGF-β1 levels with T2DN susceptibility (T2DN vs. control)

**Fig. 3:**
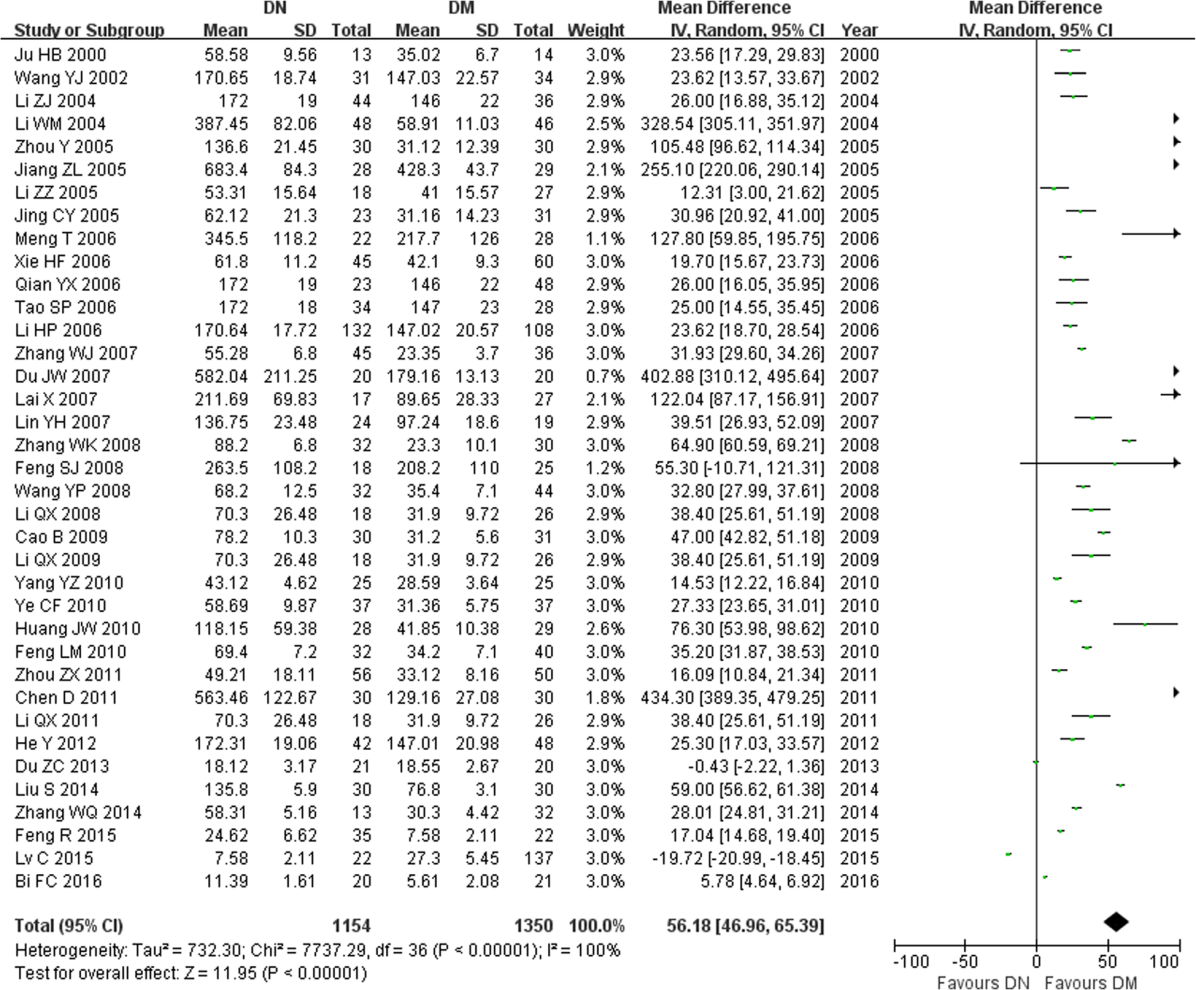
Association of TGF-β1 levels with T2DN susceptibility (T2DN vs. T2DM)

In this study, we found that the serum TGF-β1 level in the T2DM group was higher than in the normal control group (MD = 17.30, 95% CI: 12.69–21.92, *P <* 0.00001; Table 3 and Fig. 1). The serum TGF-β1 level in the T2DN group was higher than that in the normal control group (MD = 70.03, 95% CI: 60.81–79.26, *P <* 0.00001; Table 3 and Fig. 2). The serum TGF-β1 level in the T2DN group was higher than in the T2DM group (MD = 56.18, 95% CI: 46.96–65.39, *P <* 0.00001; Table 3 and Fig. 3). The serum TGF-β1 level in T2DM patients with microalbuminuria was increased compared to that in T2DM patients with normoalbuminuria (MD = 22.78, 95% CI: 16.88–28.68, *P <* 0.00001; Table 3). Furthermore, the serum TGF-β1 level in T2DM patients with macroalbuminuria was increased compared to that in T2DM patients with microalbuminuria (MD = 28.47, 95% CI: 21.28–35.66, *P <* 0.00001; Table 3).

**Table 3:** Meta-analysis of the association of TGF-β1 levels with T2DN risk in Chinese population

Contrasts	Studies number	Q test *P* value	Model selected	MD (95% CI)	*P*
DM vs. Control	38	<0.00001	Random	17.30(12.69,21.92)	<0.00001
DN vs. Control	44	<0.00001	Random	70.03 (60.81,79.26)	<0.00001
DM vs. DN	37	<0.00001	Random	56.18 (46.96,65.39)	<0.00001
Microalbuminuria VS. Normoalbuminuria	26	<0.00001	Random	22.78(16.88,28.68)	<0.00001
Macroalbuminuria VS. Microalbuminuria	24	<0.00001	Random	28.47 (21.28,35.66)	<0.00001

### Association between the TGF-β1 T869C gene polymorphism and T2DN susceptibility in Chinese population

**Fig. 4:**
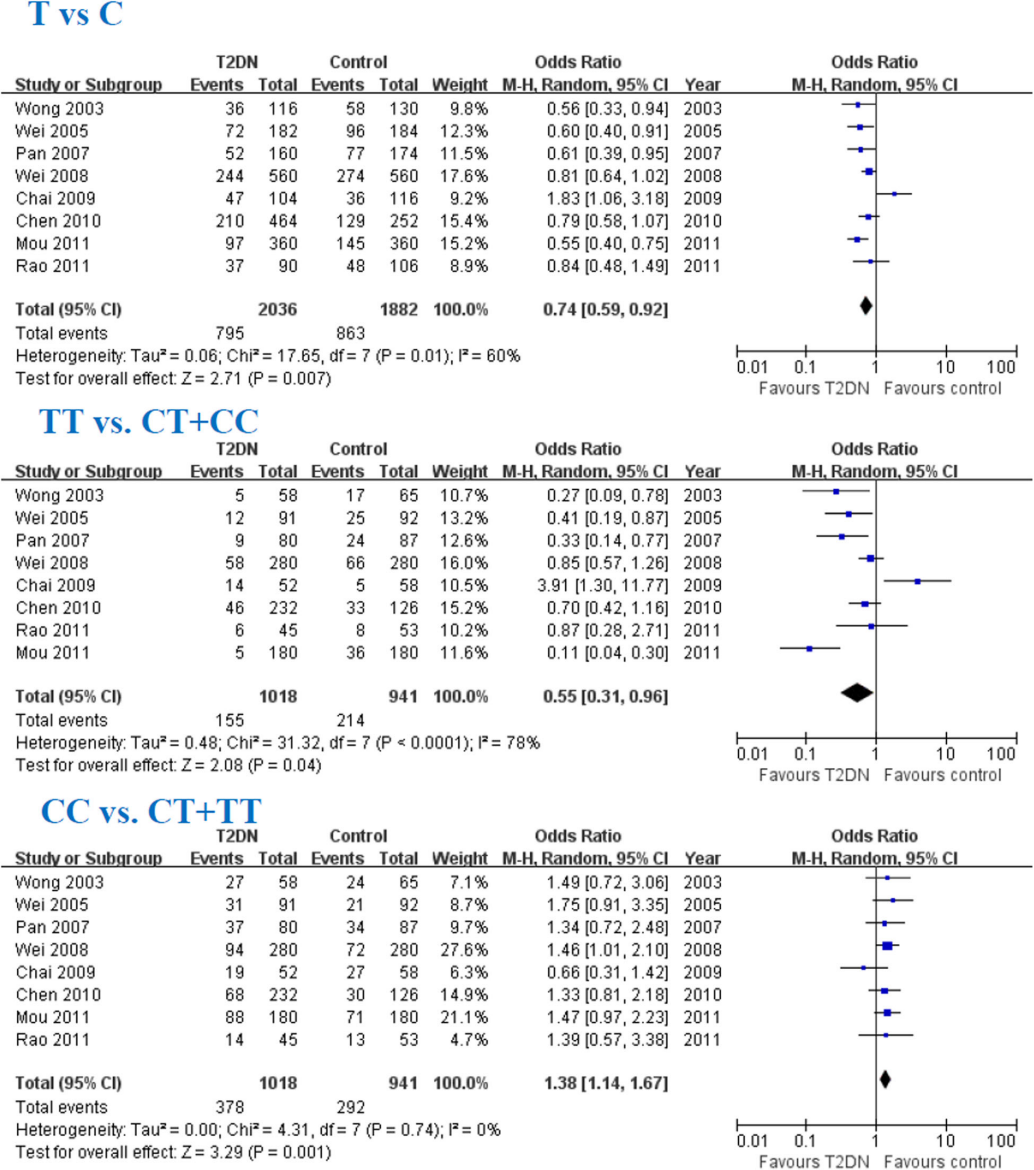
Association of TGF-β1 T869C CC genotype with DN susceptibility

In this meta-analysis, the TGF-β1 T allele, TT allele and CC genotype were associated with T2DN susceptibility in Chinese population (T: OR = 0.74, 95% CI: 0.59–0.92, *P* = 0.007; TT: OR = 0.55, 95% CI: 0.31–0.96, *P* = 0.04; CC: OR = 1.38, 95% CI: 1.14–1.67, *P* = 0.001; Fig. 4 and Table 4).

**Table 4:** Meta-analysis of the association of TGF-β1 T869C gene polymorphism with T2DN risk in Chinese population

Genetic contrasts	Studies number	Q test *P* value	Model selected	OR (95% CI)	*P*
CC vs. CT + TT	8	0.74	Fixed	1.38 (1.14,1.67)	0.001
TT vs. CT + CC	8	<0.00001	Random	0.55 (0.31,0.96)	0.04
T vs. C	8	0.01	Random	0.74 (0.59,0.92)	0.007

### Evaluation of publication bias

**Fig. 5:**
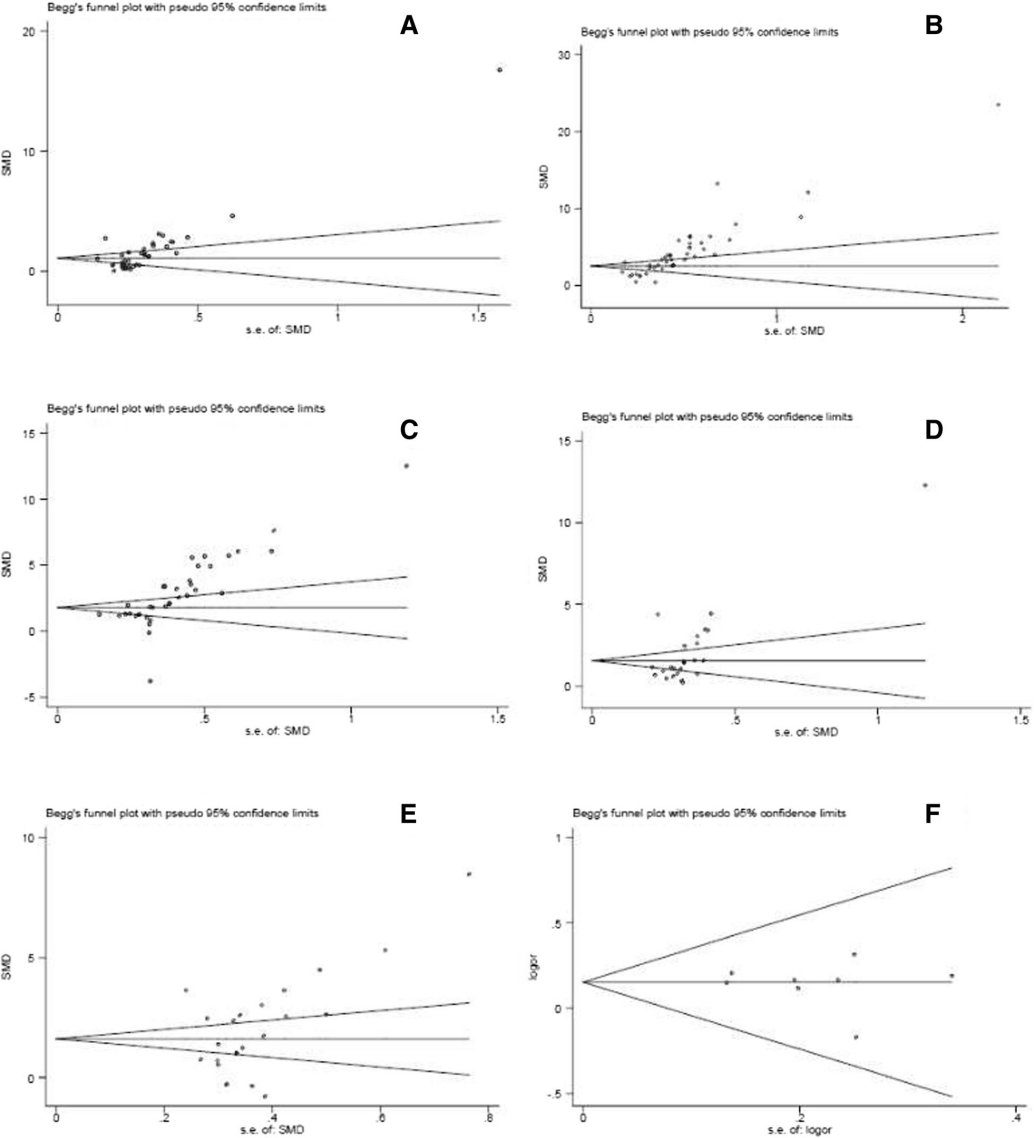
Publication bias. **a** DM vs. control. **b** DN vs. control. **c** DN vs. DM. **d** microalbuminuria vs. normoalbuminuria. **e** macroalbuminuria vs. microalbuminuria. **f** the association of the TGF-β1 T869C gene polymorphism with T2DN susceptibility in Chinese population

There were publication biases for DM vs. control (Egger *P* = 0.001, Begg *P* = 0; Fig. 5a), DN vs. control (Egger *P* = 0, Begg *P* = 0; Fig. 5b), DN vs. DM (Egger *P* = 0, Begg *P* = 0; Fig. 5c), microalbuminuria vs. normoalbuminuria (Egger *P* = 0.021, Begg P = 0; Fig. 5d), macroalbuminuria vs. microalbuminuria in Chinese population (Egger *P* = 0.051, Begg *P* = 0.042; Fig. 5e). Interestingly, there was no publication bias for the association of the TGF-β1 T869C gene polymorphism with T2DN susceptibility in Chinese population (Egger *P* = 0.627, Begg *P* = 1.000; Fig. 5f).

## Discussion

TGF-β1 can stimulate the transcription of extracellular matrix (ECM) proteins, and high levels of TGF-β1 are associated with ECM accumulation, fibrosis, and glomerulosclerosis. Glomerulosclerosis is one of most important characteristics of patients with T2DN. In this study, we performed the meta-analysis in Chinese population and found that serum levels of TGF-β1 in the T2DM group were higher than those in the normal control group. The serum TGF-β1 level in the T2DN group was higher than that in the normal control group or the T2DM group. Indeed, the levels of TGF-β1 in the T2DM group and the T2DN group were higher than those in the normal control group. The level of TGF-β1 in T2DN was higher than that in the other two groups. We also performed a subgroup analysis according to albuminuria levels. The serum TGF-β1 level in T2DM patients with microalbuminuria was increased over that in T2DM patients with normoalbuminuria, and the serum TGF-β1 level in T2DM patients with macroalbuminuria was increased over that in T2DM patients with microalbuminuria. This indicated that the more urine protein is, the more severe the kidney disease becomes.

Qiao et al. [75] conducted a meta-analysis based on 26 studies with 1968 cases and 2100 controls to evaluate the association between the levels of serum TGF-β1, and urinary TGF-β1 in patients with DM or diabetic nephropathy (DN). They reported that the levels of serum and urinary TGF-β1 were significantly increased in T2DM and T2DN. Mou et al. [76] assessed 9 reports that included 264 patients and 227 healthy controls in a meta-analysis to study the relationship between serum TGF-β1 levels and the risk of diabetic nephropathy. Their study indicated that increased serum TGF-β1 levels in DM patients were associated with a high risk of renal involvement. The results from Qiao et al. and Mou et al. indicated that serum and urinary TGF-β1 were significantly increased in DM and DN. Our meta-analysis included 45 reports to study the relationship between TGF-β1 level and T2DN risk in Chinese population. Our study concludes that high levels of TGF-β1 are associated with the susceptibility to T2DM, T2DN, and the progression of proteinuria in T2DN patients in Chinese population.

The association of the TGF-β1 T869C gene polymorphism with the risk of T2DN in Chinese population was also assessed. In this meta-analysis, we found that TGF-β1 T allele, and TT genotype were protective factors against the onset of T2DN in Chinese population and CC genotype was a risk factor for the susceptibility of T2DN in Chinese populations. There was no publication bias for this meta-analysis. The results might be robust to some extent. However, there were only eight studies included into for this meta-analysis in Chinese population and more number of studies should be conducted to confirm the validity of these conclusions in the future.

In a previous study, Jia et al. [77] conducted a meta-analysis to evaluate the impact of the TGF-β1 T869C gene polymorphism on DN, and reported that the TGF-β1 T869C gene polymorphism was associated with an elevated risk of DN disease. However, this notable association was observed only in T2DM patients. Zhou et al. [78] conducted a meta-analysis and indicated that the TGF-β1 CC genotype was associated with T2DN risk, and that the TGF-β1 T allele and the CC genotype were associated with the susceptibility to T2DN. In this meta-analysis, we firstly conducted the meta-analysis in Chinese population and observed that the TGF-β1 T allele, TT genotype and CC genotype are associated with the susceptibility to T2DN in Chinese population. However, more studies are also needed to confirm this in the future.

The conclusions of our meta-analysis are limited because of the nature of the studies we analyzed. The studies themselves had several limitations, such as publication bias (most of the included studies from Chinese populations), heterogeneity of enrolled cases, small sample sizes, varying levels of plasma protein in different studies and different samples, and different timelines. In this meta-analysis, we conducted a subgroup analysis to delete any study with small sample size (less than 100), and we found that in the meta-analysis of only the larger sample studies, the CC genotype was associated with T2DN susceptibility (data not shown). However, the TGF-β1 T869C gene polymorphism was not associated with T2DN susceptibility in the meta-analysis that included small sample size studies (data not shown). In this study, we also found that there were publication biases among the recruited investigations for the relationship between serum TGF-β1 levels and the risk of T2DN, and for the relationship between the TGF-β1 T869C gene polymorphism and the risk of T2DN.

## Conclusions

In conclusion, this study indicated that the serum TGF-β1 level in T2DM patients with microalbuminuria was significantly increased over that in T2DM patients with normoalbuminuria in Chinese population. The serum TGF-β1 level in T2DM patients with macroalbuminuria was significantly increased over that in T2DM patients with microalbuminuria in Chinese population. Furthermore, the TGF-β1 T allele, TT genotype and CC genotype are associated with the susceptibility to T2DN in Chinese population. However, more association studies are required to confirm the relationships.

## Abbreviations

DM: Diabetes mellitus
DN: diabetic nephropathy
ECM: extracellular matrix
T2DN: type 2 diabetic nephropathy
TGF-β1: transforming growth factor-β1
